# Nanotechnology-Based Cosmeceuticals

**DOI:** 10.1155/2014/843687

**Published:** 2014-05-22

**Authors:** Alka Lohani, Anurag Verma, Himanshi Joshi, Niti Yadav, Neha Karki

**Affiliations:** ^1^School of Pharmaceutical Sciences, IFTM University, Moradabad, Uttar Pradesh 244102, India; ^2^GRD Institute of Management and Technology, Dehradun, Uttarakhand 248009, India; ^3^Institute of Biotechnology, Patwadangar, Nainital, Uttarakhand 263128, India

## Abstract

Cosmeceuticals are the fastest growing segment of the personal care industry, and a number of topical cosmeceutical treatments for conditions such as photoaging, hyperpigmentation, wrinkles, and hair damage have come into widespread use. In the cosmeceutical arena nanotechnology has played an important role. Using new techniques to manipulate matter at an atomic or molecular level, they have been at the root of numerous innovations, opening up new perspectives for the future of cosmeceutical industry. Nanotechnology-based cosmeceuticals offer the advantage of diversity in products, and increased bioavailability of active ingredients and increase the aesthetic appeal of cosmeceutical products with prolonged effects. However increased use of nanotechnology in cosmeceuticals has raised concern about the possible penetration of nanoparticles through the skin and potential hazards to the human health. This review outlines the different nanoparticles used in various classes of cosmeceuticals, nanotechnology-based cosmeceutical products present in the market, and the potential risk caused by nanoparticles on exposure and recent regulatory steps taken to overcome them.

## 1. Introduction

Cosmetics are defined by the FDA as “articles intended to be applied to the human body or any part thereof for cleansing, beautifying, promoting attractiveness, or altering the appearance” [1]. FDA does not have the legal authority to approve cosmetics before they go on the market. However, cosmetics must be safe for consumers and properly labeled. Companies and individuals who market cosmetics have a legal responsibility for the safety and labeling of their products [2]. The word “cosmeceutical” is used to define a product that fits the niche between a drug and cosmetics [3]. It is used in the professional skin care arena to describe a product that has measurable biological action in the skin, like a drug, but is regulated as a cosmetic since it claims to affect appearance [4]. Cosmeceuticals are not categorized by the FDA, but this term is used by skin scientists, physicians, and skin care professionals, to encourage the consumers to continue buying cosmetic products especially antiaging and sunscreen products, marketed by many manufacturers with scientific claims and natural positioning as a way to emphasize that using these products is not only necessary but also natural. Cosmeceuticals are the fastest growing segment of the personal care industry [5]. Cosmeceutical formulations now have expanded from skin to body to hair and a number of topical cosmeceutical treatments for conditions such as photoaging, hyperpigmentation, wrinkles, and hair damage have come into widespread use [6]. Recent researches focusing on cosmeceutical products highlighted strong growth perspectives in the coming years. According to them expanding at a rapid compound annual growth rate of 7.7%, the global cosmeceutical market will reach $31.84 billion by 2016 [7]. The global cosmeceutical market offers huge potential among the Asian countries, such as Japan, China, and India which are set to attract major players in the future. Japan has already made a remarkable position in the global cosmetics market and its position in the cosmeceutical segment is effectively improving [7]. A report, “Cosmeceuticals market to 2018,” forecasted that the global cosmeceuticals market will reach $42.4 billion by 2018 [8].

Among the technologies used to develop elegant and effective cosmeceuticals, nanotechnology finds special place. In the cosmetic arena it is believed that the smaller particles are readily absorbed into the skin and repair damage easily and more efficiently [9]. Incorporation of nanotechnology in cosmeceuticals is aimed at making incense of perfumes last longer, sunscreens to protect the skin, antiaging creams to fight back the years, and moisturizers to maintain the hydration of skin. Some of the nanotechnology-based innovations are nanoemulsions (which are transparent and have unique tactile and texture properties), nanocapsules (which are used in skin care products), nanopigments (that are transparent and increase the efficiency of sunscreen products), liposome formulations (which contain small vesicles consisting of conventional cosmetic materials that protect oxygen or light sensitive cosmetic ingredients), niosomes, nanocrystals, solid lipid nanoparticles, carbon nanotubes, fullerenes, and dendrimers. The primary advantages of using nanoparticles in cosmeceuticals include improvement in the stability of cosmetic ingredients (e.g., vitamins, unsaturated fatty acids, and antioxidants) by encapsulating within the nanoparticles; efficient protection of the skin from harmful ultraviolet (UV) rays; aesthetically pleasing products (e.g., in mineral sunscreens, using smaller particles of active mineral allows them to be applied without leaving a noticeable white cast); targeting of active ingredient to the desired site and controlled release of active ingredients for prolonged effect [10, 11].

## 2. Nanoparticles in Cosmeceuticals

### 2.1. Liposomes

**Figure 1:**
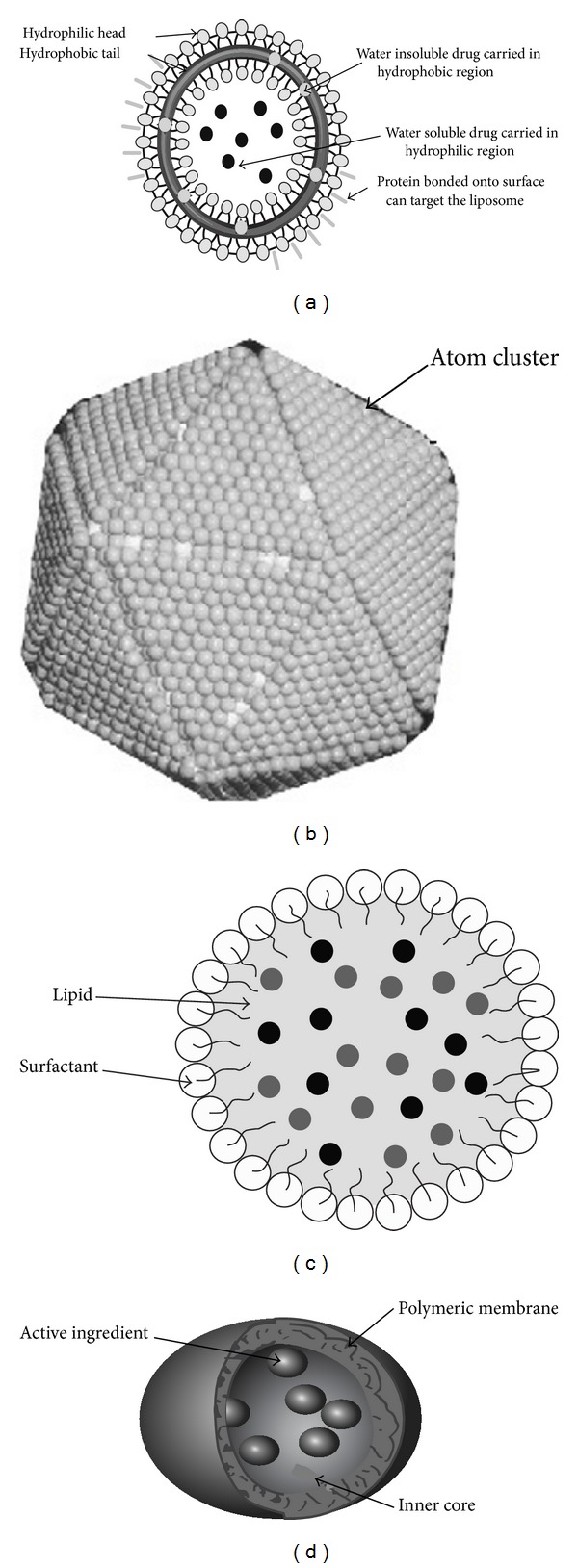
Different types of nanoparticles. (a): liposome showing a phospholipid bilayer surrounding an aqueous interior, (b): nanocrystal, (c): solid lipid nanoparticle [12], and (d): nanocapsule with different drug-loading modalities [13].

Bangham published the first paper on liposomes in 1963, and it was in the early 1980s that Mezei and Gulasekharam reported the efficacy of liposomes in topical drug delivery [14, 15]. Liposomes are spherical, self-closed vesicles of colloidal dimensions, in which phospholipid bilayers sequester part of the solvent, in which they freely float, into their interior (Figure 1). Liposomes typically vary in size between 20 nm and a few hundred micrometers [16]. Liposomes are used in a variety of cosmeceuticals because they are biocompatible, biodegradable, nontoxic, and flexible vesicles and can encapsulate active ingredients easily. Liposomes have an ability to protect the encapsulated drug from external environment and are suitable for delivery of hydrophobic and hydrophilic compounds [16]. These characteristics make them ideal candidate for the delivery of vitamins and other essential molecules to regenerate the epidermis [17]. One of the main ingredients of liposome is Phosphatidylcholine which has been used in skin care products (moisturizer, lotions, creams, etc.) and hair care products (shampoo, conditioner) due to its softening and conditioning properties. Several active ingredients (e.g., vitamins A, E, and K) and antioxidants (e.g., Carotenoids, lycopene, and CoQ10) have been incorporated into liposomes which increases their physical and chemical stability when dispersed in water. Lipophilic compounds such as cholesterol and ceramides have been used in topical skin creams for many years, because they are the lipids found in normal skin tissue, and are easily incorporated into liposomes to improve skin hydration and to make the skin texture softer and smoother. “Capture” was the first liposomal antiageing cream launched by Dior in 1986 [18].

### 2.2. Nanocapsule

The potential dermatological use of nanocapsules was investigated when the first nanocapsule-based cosmetic product was launched by the French company L'Oreal in 1995 in order to improve the impact of their cosmetics [19]. The term nanocapsule is used for vesicular systems that are made up of a polymeric membrane in which an inner liquid core is encapsulated at the nanoscale level (10 nm to 1000 nm) (Figure 1) [20].

### 2.3. Solid Lipid Nanoparticles

Solid lipid nanoparticles (SLNs) (Figure 1) are submicron colloidal carriers whose size ranges from 50 to 1000 nm and are composed of physiological lipid, dispersed in water or in aqueous solution of surfactant [12]. SLNs are popular in cosmeceuticals because of various advantages: these are composed of physiological and biodegradable lipids that exhibit low toxicity; the small size of SLNs ensures close contact with the stratum corneum and increases the penetration of active ingredients through the skin; SLNs provide occlusive properties that result in increased skin hydration [21]. The products Nano Repair Q10 cream and Nano Repair Q10 Serum (Dr. Kurt Richter Laboratorien GmbH, Berlin, Germany) introduced to the cosmetic market in October 2005 revealed the success of lipid nanoparticles in the antiageing field [22]. It has been found that SLNs possess characteristics of physical UV blockers on their own, thus offering the choice for developing a more effective sunscreen system with reduced side effects [23]. In an* in vivo *study it has been shown that skin hydration increases by 31% after 4 weeks by the addition of 4% SLNs to a conventional cream [24]. SLNs are also advantageous as topical vehicle for perfumes. By incorporating perfumes/fragrances in SLNs, the release can be slowed down to provide prolonged effect [25].

### 2.4. Nanocrystals

Nanocrystals are aggregates composed of several hundreds to thousands of atoms that combine into a cluster and are in the size range of 10–400 nm used for the delivery of poorly soluble actives (Figure 1) [26]. Nanocrystals appeared first in the cosmeceutical market in 2000 by Juvena with the product Juvedical having rutin [27]. In a study it was observed that, compared to the water-soluble rutin glucoside (rutin with attached glucose), the nanocrystal formulation of original rutin molecule possesses 500 times higher bioactivity [28]. A rutin nanosuspension with 5% rutin as nondissolved nanocrystals was applied to the skin of human volunteers and compared to a 5% solution of a water-soluble rutin glucoside regarding photoprotection of the skin. In the aqueous nanosuspension, the solubility of rutin was 500 times lower as compared to the water-soluble derivative. It was observed that, despite the 500 times lower concentration of dissolved rutin in the water phase of the nanocrystal suspension, the nanosuspension was about 25% more effective in photoprotection and the concentration of actives formulated as nanocrystals in the skin were much higher compared to water-soluble derivative or using the active in normal powder form.

### 2.5. Dendrimers

**Figure 2:**
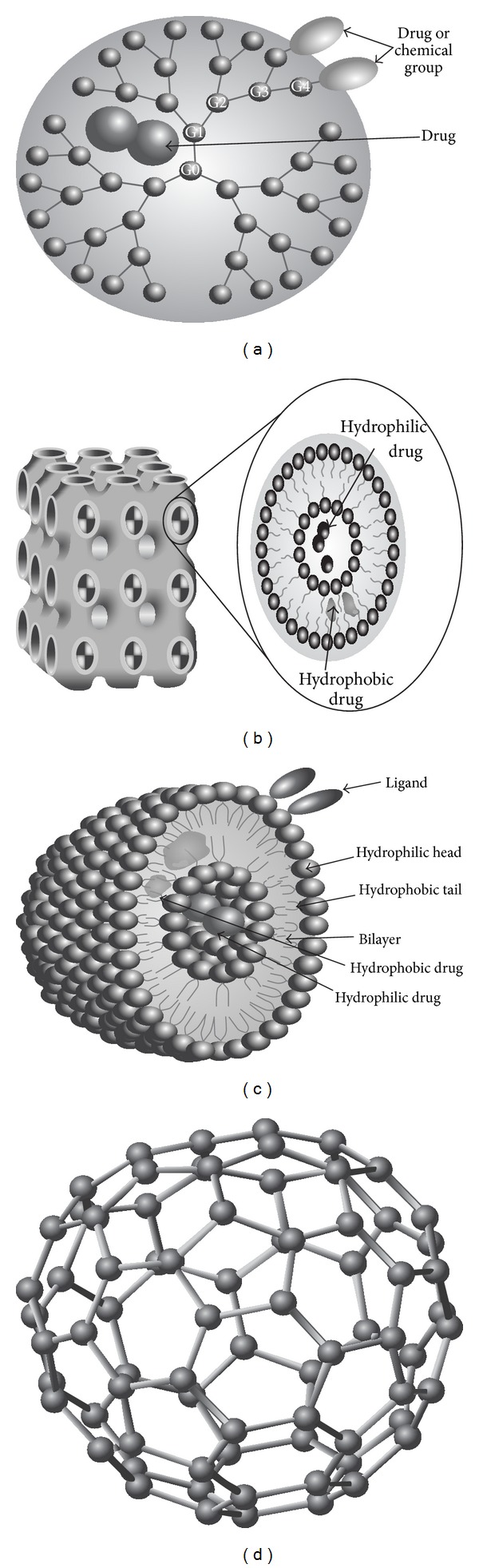
Different types of nanoparticles. (a): dendrimer with its different drug-loading modalities, (b): cubosome and its membrane composition with different drug-loading modalities. (c): niosome and its internal synthetic surfactant surrounding drug, and (d): fullerene [13].

Dendrimers are organic chemical entities with a semipolymeric tree-like structure (Figure 2). The terminals of the branches provide a rich source of nanoparticles surface functionality. Their dimensions are extremely small, having diameters in the range of 2 to 10 nm [29]. Dendrimers are an exciting new class of macromolecular architecture and an important component in the area of nanotechnology-based cosmeceuticals to treat varieties of skin conditions. L'Oreal, Unilever, and The Dow Chemical Company have several patents for the application of dendrimers in hair care, skin care, and nail care products [30]. A patent on cosmetic formulation containing carbosiloxane dendrimer claimed that it can provide good water resistance, sebum resistance, glossiness, tactile sensation, and/or adhesive properties to the hair and/or skin [31].

### 2.6. Nanogold and Nanosilver

Gold and silver nanoparticles have been studied as a valuable material in cosmeceutical industry for their strong antibacterial and antifungal properties. These particles are widely used in cosmeceutical products like deodorant, face pack, antiaging cream, and so forth. An ointment containing silver nanoparticle was claimed to have antibacterial activity and can be used for skin inflammation and skin wound disinfection [32]. A study conducted by French scientist Dr. Philippe Walter and his team, published in ACS Nanoletters, describes the synthesis of fluorescent gold nanoparticles inside human hair. It involved soaking white hairs in a solution of a gold compound. The hairs turned pale yellow and then darkened to a deep brown. Using an electron microscope, the scientists confirmed that the particles were forming inside the hair's central core cortex. The color remained even after repeated washings [33].

### 2.7. Cubosomes

Cubosomes are discrete, submicron, nanostructured particles of bicontinuous cubic liquid crystalline phase (Figure 2) [34]. Recent research activities on the use of cubosome in personal care product areas varied from skin care to hair care and antiperspirants. The number of researches in association with cosmetic companies like L'Oreal and Nivea is trying to use cubosome particles as oil-in-water emulsion stabilizers and pollutant absorbents in cosmeceuticals [35–38].

### 2.8. Niosomes

Niosomes are nonionic surfactant vesicles devised by using nonionic surfactants (Figure 2) [39]. These vesicles possess high entrapment efficiency, improved chemical stability, and enhanced penetration, as well as lower production cost as compared to liposomes. In morphology, a niosome is a nanostructure with 100 nm to 2 *μ*m in diameter, whose center is an aqueous cavity enveloped by layers of nonionic surfactant in lamellar phase [13]. These have been evaluated as vesicular carriers for variety of drugs and cosmetics topically. Niosomes are found to be efficient in topical delivery of active ingredients as they can enhance residence time of the active ingredients in the stratum corneum as well as epidermis and also reduce the system absorption [39]. By using niosomes, targeted delivery can also be achieved as the active ingredient is directly delivered to the specific site where therapeutic effect is desired [40].

### 2.9. Fullerene

Other nanoscale materials such as carbon fullerene have been used in some cosmetic products because of their antioxidative properties. They display potent scavenging capacities against radical oxygen species and they have been considered for their use in the preparation of skin rejuvenation cosmeceutical formulations [41]. These structures are comprised of carbon rings and contain odd-numbered (like Pentagon and heptagon) carbon rings, conferring a three dimensional spherical shape [42]. These structures have thus been called fullerenes or “Bucky Balls” (Figure 2). Fullerenes are highly hydrophobic and thus are not soluble in aqueous solutions, which initially limited their applications, but the use of surfactants or surface modifications has increased the ability of fullerenes to solubilize in water and brought more attention to their potential pharmaceutical uses [43].

## 3. Major Classes of Nanocosmeceuticals

### 3.1. Moisturizers

Stratum corneum is the primary barrier of the skin whose main purpose is to keep inside in and outside out. Water from the stratum corneum gets evaporated quickly leading to dehydration. This dehydration of skin can be averted by using moisturizers which provide flexibility to the skin. When moisturizers are applied to the skin, a thin film of humectant is formed which retains moisture and gives better appearance to the skin. Liposomes, nanoemulsions, SLNs are widely used moisturizing formulations because of their prolonged effects. These are considered to be the most useful product for the management of various skin conditions (e.g., atopic dermatitis, psoriasis, and pruritus).

### 3.2. Sunscreens

Sunscreens are widely used to protect the skin from harmful effects of sun rays on exposure. Zinc oxide (ZnO) and titanium dioxide (TiO_2_) are the most effective approved mineral-based ingredient which protects the skin from sun damage. This mineral forms a materialistic barrier on the skin, reflects UVA and UVB rays from penetrating down to the deeper layers of skin, and is less irritating [45]. The main drawback of traditional or conventional sunscreen is that, when applied, it leaves a white chalky layer on the skin [46]. This is where nanoparticles come in. Improved sunscreens are just one of the many innovative uses of nanotechnology. Sunscreen products using nanoparticles of ZnO or TiO_2_ are transparent, less greasy, and less smelly and have increased aesthetic appeal.

### 3.3. Antiaging Products

Chemical products, pollution, stress, irradiation from infrared (IR) and ultraviolet (UV) sources, and abrasion are involved in skin aging. Collagen plays an important role in skin rejuvenation and wrinkle reversal effect. The quantity of collagen in the skin decreases along with age. The aging of the skin manifests itself in many ways: drying out, loss of elasticity and texture, thinning, damaged barrier function, appearance of spots, modification of surface line isotropy, and, finally, wrinkles. Most of the cosmeceuticals have been developed with claims of antiwrinkle and firming, moisturizing and lifting, and skin toning and whitening activity. Antiaging products are the main cosmeceuticals in the market currently being made using nanotechnology. L'Oreal has employed nanotechnology in products such as Revitalift antiwrinkle cream which contains nanosomes of Pro-Retinol A, and claims that it instantly retautens the skin and reduces the appearance of wrinkles [47]. Application of retinol can increase epidermal water content, epidermal hyperplasia, and cell renewal while enhancing collagen synthesis [48]. Retinol also interferes with melanogenesis and inhibits matrix metalloproteinases, which are involved in collagen breakdown. The clinical benefits include a reduction in the appearance of fine lines and wrinkles and lightening of lentigines [49]. Lancôme introduces Hydra Zen Cream to renew the skin's healthy look which contains nanoencapsulated Triceramide [50].

### 3.4. Hair Care

**Figure 3:**
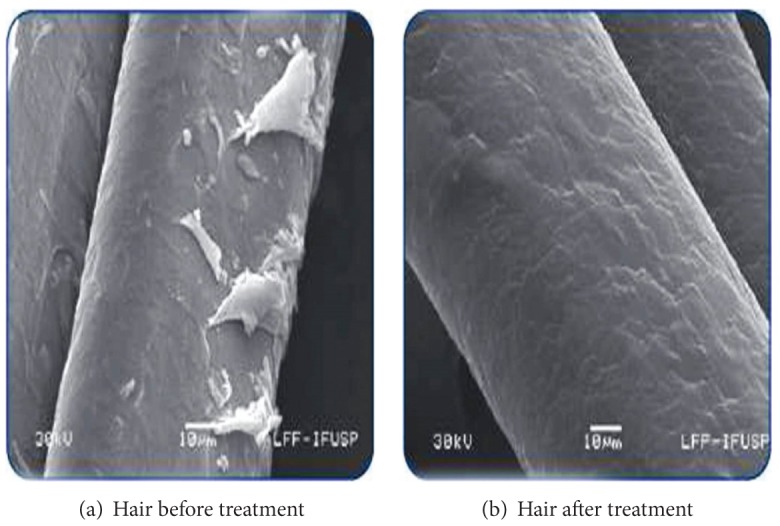
Effect of sericin nanoparticles on hair cuticle. Increased hair gloss (b) obtained in damaged hair (a) after treating with sericin nanoparticles [44].

Hair care is another promising field for nanotechnology. Companies are using nanotechnology in hair care products and research is ongoing to discover the ways of how nanoparticles can be used to prevent hair loss and to maintain shine, silkiness, and health of hairs. Unlike ordinary hair straightening products nanoemulsion in hair cosmetics does not destroy the outer structure of the hair fibers, called cuticles, to penetrate into the hair strands [51]. Sericin (composed of cationic sericin nanoparticles) is an active area of hair cosmeceuticals. Studies have shown that sericin nanoparticles in hair cosmeceuticals easily adhere to the surface of hair seal and treat the damaged cuticles (Figure 3) [44].

### 3.5. Skin Cleanser

The skin is covered with a hydrolipid film that, depending on the area of the body, comprises secretions from sebaceous glands and from apocrine and eccrine sweat glands. Decomposition products from corneocytes and cornification (cellular debris and stratum corneum lipids) in the process of being shed are also present. This film provides a natural defense against pathogenic organisms but also attracts dirt and pollutants from the environment. Sometimes the microorganisms present on the skin surface act on components of the surface film and create undesirable by-products, such as those resulting from the metabolism of compounds found in apocrine sweat that create body odor [52]. Thus, periodic cleansing to remove debris, dirt, and odor is essential to maintain skin health. Cleansing is also necessary to remove soil (which may include bacteria) from the skin surface that is acquired by incidental contact or by intentional application (medications or makeup and other cosmetic products). Silver nanoparticles are used as skin disinfectant and decontamination. Nano Cyclic Inc. produces Nano Cyclic cleanser pink soap which is a scientifically balanced blend of nanosilver and natural ingredients and claims that it kills harmful bacteria and fungi, fights acne, and diminishes age spots and sun damaged skin [53].

### 3.6. Lip Care

Lip care is another promising class of cosmeceuticals. Different nanoparticles can be incorporated into lipstick and lip gloss which will soften or soothe the lips by preventing transepidermal water loss. Korea Research Institute of Bioscience and Biotechnology holds a patent that described that it is possible to prepare pigments exhibiting wide range of colors using gold or silver nanoparticles by mixing in various compositional ratios and whose color can be maintained for a long period of time [54]. Silica nanoparticles used in lipsticks improve the homogenous distribution of pigments. Once applied, they prevent the pigments from migrating or bleeding into the fine line of lips [55].

### 3.7. Nail Care

Nanotechnology-based nail cosmeceuticals have various advantages over conventional products. A study revealed that nail paints having nanosized particles improve toughness, mar resistance, and impact resistance of the mammalian nails [56]. Nano Labs Corp. (a nanotechnology research and development company) was awarded a provisional patent for its original nanonail polish and lacquer having advantages that it dries to a very hard state, resists shock, cracking, scratching, and chipping and its elasticity offers superior ease of application without cracking [57]. One of the new strategies which may have great potential in the cosmeceuticals is the incorporation of nanoparticles having antifungal activity (like silver and metal oxide nanoparticles) in nail polish to treat fungal toenail infections.

A review on various nanotechnology-based cosmeceutical products in the market and patents have been tabulated in Tables 1 and 2.

## 4. Exposure to Nanoparticles

Industrial use of nanoparticles has created new opportunities, but it also presents some risks and uncertainties. Increasing production and use of nanomaterials results in an increasing number of workers and consumers exposed to nanomaterials. This shows that there is greater need for information on their exposure routes. Human routes of exposure to nanoparticles are inhalation, ingestion, and dermal routes [58]. Inhalation is the most common route of exposure to airborne nanoparticles [59]. Workers may inhale nanoparticles while production or consumers may inhale on the use of aerosolized cosmeceuticals (deodorant, perfumes, etc.). The deposition of nanoparticles in the respiratory system depends on their interactions with respiratory epithelium membrane. Nanoparticles may travel via the nasal nerves to the brain (transsynaptic transport after inhalation through the olfactory epithelium) and gain access to the nervous system [60]. Because of their size, these nanoparticles can easily gain access to the blood stream inhalation or skin and from there they are transported to the various organs [58]. Ingestion may occur from unintentional hand to mouth transfer of nanoparticles or from those cosmeceuticals that are applied near mouth or lips (e.g., lip color, lip gloss). Large fractions of nanoparticles rapidly pass out of the body after ingestion, but a small fraction may be taken up by the body which migrates into the different organs [61]. The other route of exposure of nanoparticles into the systemic circulation is dermal absorption. Majority of cosmeceuticals are applied to the skin. Three pathways of penetration across the skin have been identified: intercellular, transfollicular, and transcellular [62].

## 5. Skin Penetration of Nanoparticles

**Figure 4:**
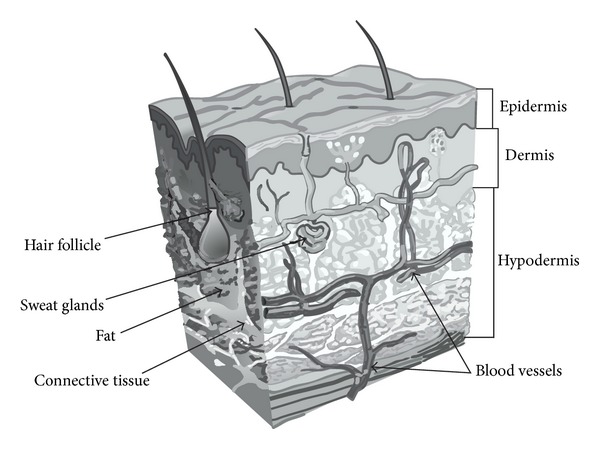
Human skin layers.

**Figure 5:**
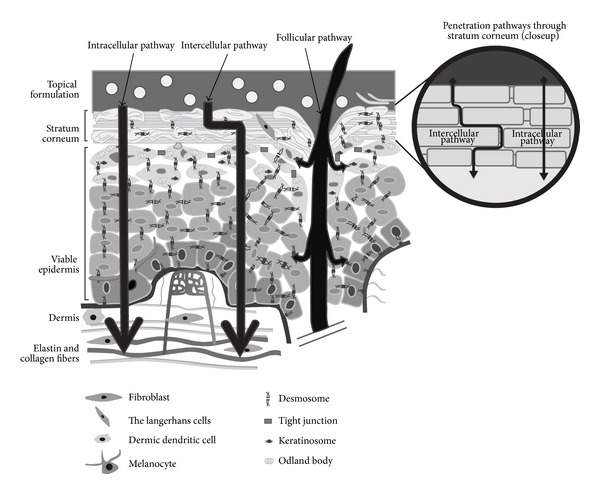
Skin penetration pathways (intracellular, intercellular, and follicular) by which a molecule can cross the stratum corneum [64].

The skin is the largest organ of the body. Human skin is made up of three layers (Figure 4): the epidermis (the outermost layer of skin), the dermis (contains tough connective tissue, hair follicles, and sweat glands), and the hypodermis (made up of fat and connective tissue). The epidermis is divided into several layers and its outermost layer, the stratum corneum, is responsible for the barrier function of the skin due to its lipophilicity and high cohesion between cells [63]. Passive routes by which a molecule can cross the stratum corneum are intercellular, transcellular, and appendageal routes (Figure 5) [64].

A variety of cosmeceutical products having nanoparticles are in the market that are applied to the skin and concerns have been raised regarding the potential dangers which may occur on their skin penetration. The transport of nanoparticles through the skin is related to the nature and physicochemical properties of the nanoparticles and vehicles, the nature of the substance, and the conditions of the skin [65]. Nanoparticles can be divided into two groups: (1) soluble and/or biodegradable nanoparticles (e.g., liposomes and nanoemulsion); (2) insoluble and/or nonbiodegradable nanoparticles (e.g., TiO_2_, fullerenes, and quantum dots). Dermal absorption of nanoparticles does not occur readily but can take place under certain conditions. Although cosmetic products are meant to be used on normal skin, it is known that they are also applied on nonhealthy skin. In such conditions the barrier properties of skin may be impaired. Most of the study reported that nanoproducts applied to the skin only penetrate through hair follicular openings and skin pores, with minimal amount being found below the stratum corneum [66].

Research on the fate of these nanoparticles when applied to mammalian skin by employing laser scanning confocal microscopy to see whether fluorescently tagged particles (20 to 200 nm) were absorbed into the skin showed that nanoparticles contacting intact or partially damaged skin cannot penetrate skin barrier and do not reach the viable cells of the epidermis or beyond and hence proved that the nanotopical delivery systems are useful and safe for cosmeceuticals [67]. In a study on dermal absorption of ZnO nanoparticles from sunscreen applied to humans at the beach, Gulson et al. revealed that zinc from ZnO particles in sunscreen penetrates healthy skin and is observed in blood and urine. Whether the Zn was present as particles or soluble Zn ion was unknown at that stage [68]. A review on the use of nanoparticles in personal care products done by the Environmental Working Group, a US-based NGO, concluded after peer review of more than 400 documents that: “zinc and titanium-based formulations are among the safest, most effective sunscreens on the market based on available evidence” and of 16 studies on skin absorption, “nearly all showing no absorption of smallscale zinc and titanium sunscreen ingredients through healthy skin” [69]. The controversy has intensified, with numerous studies reporting that they do not cross the skin barrier, whilst others lead us to suspect new risks to humans, though without any of them providing a definitive answer. A review of percutaneous absorption studies of TiO_2_ and ZnO nanoparticles has been shown in Table 3 [70–77].

Continued research is required to evaluate the behavior of nanoparticles, including whether the nanoparticles remain on the surface of skin and/or stratum corneum or absorbed into the blood stream to reach different organs.

## 6. Toxicity of Nanoparticles

Nanoparticles from various cosmeceutical products applied on skin can have toxic effects if reaching to blood stream. A research on toxicity of TiO_2_ nanoparticles demonstrated that when nano-sized TiO_2_ administered subcutaneously to pregnant mice, they transferred to the offspring and result in brain damage and reduced sperm production in male offspring [78]. Various researches have shown that TiO_2_ nanoparticles can produce free radicals and cause cell toxicity in test tube studies, when exposed to UV light [79, 80]. Studies have shown that cobalt-chromium nanoparticles (29.5 nm in diameter) can destroy human fibroblast cells across an intact cellular barrier. If nanoparticles are inhaled and eaten accidentally or absorbed through skin, they could cause skin and lung damage and organ toxicity or can harm unborn children [81]. Silver nanoparticles are used in cosmeceuticals for their antimicrobial activity. Concentration of silver that is lethal for bacteria is also lethal for both keratinocytes and fibroblasts [82]. The cosmeceutical industry debates that consumer risks are low, as there is no evidence that nanoparticles from the product penetrate healthy, intact adult skin.

## 7. Recent Advances in Nanoproduct Regulation

Recently USFDA has published an Import Alert 66-38, for skin care products labeled as antiaging creams [83]. This is because there are numerous skin care products in the market which claim that the products counteract, retard, or control the aging process. According to USFDA, A claim such as “molecules absorb and expand, exerting upward pressure to lift wrinkles upward” is a claim for an inner structural change that would usually cause a product to be a drug. FDA has stated such claims are illegal on cosmetic labeling.

In the European Union (EU), the new Cosmetic Products Regulation 1223/2009 attempts to go some way in addressing concerns over nanomaterials. According to this regulation all ingredients present as nanomaterials must be indicated on the package, from July 11, 2013, with the word “nano” [84]. The format distinguishes a nanoparticle with the suffix “nano,” so TiO_2_ becomes TiO_2_-nano [85, 86]. The regulation also requires that all marketed cosmetics and sunscreens using nanoparticles be individually tested for safety. Cosmetic products containing nanomaterials must be notified by electronic means to the commission, providing data on identification, specification, quantity, toxicological profile, safety data, and foreseeable exposure conditions. Such notification must occur six months before a cosmetic product containing nanomaterials is placed on the market [84].

## 8. Conclusion

Growth of cosmeceutical industry is increasing day by day as the cosmeceuticals market is highly diversified, with products coming from major and small manufacturers and local companies around the world. Nanotechnology represents the key technologies of the twenty-first century, offering excellent opportunities for both research and business. The rapid spread and commercialization of nanotechnology in cosmeceuticals have given rise to great technical and economic aspirations but also question about the emerging risks to health and safety of consumers. Thus, cosmeceutical products based on nanotechnology should be designed and sold in a way that fully respects the health of consumers and the environment.

## Figures and Tables

**Table 1:** Various nanotechnology-based cosmeceutical products in the market.

Product	Proposed use	Manufacturer	Marketing claims
Hydra Flash Bronzer Daily Face moisturizer	Moisturizer	Lancôme	Nanocapsules of pure vitamin E provide powerful antioxidant protection. A light touch of self-tanner ensures a natural, healthy glowing skin.

Hydra Zen Cream	Moisturizer	Lancôme	Containing Nanoencapsulated Triceramides, Hydra Zen helps restore perfect comfort and softness and renew skin's healthy look. Protected from signs of daily stress and fully hydrated, your skin is beautifully soft and smooth all day long.

Nano-In Hand and Nail Moisturizing Serum and Foot Moisturizing Serum	Moisturizer	Nano-Infinity Nanotech	Fine crystals of ZnO nanoparticles will go straight into skin tissue to prevent hand and nails from being hurt and restore skin health

Lancôme Renergie Microlift	Antiwrinkle	Lancôme	Formulated with colloidal silica and soy protein nanoparticles to provide the closest possible face-lift effect.

RevitaLift Anti-Wrinkle and Firming Face and Neck Contour Cream	Antiwrinkle	L'Oreal	The Revitalift formula is enriched with Pro-Retinol A, a powerful antiwrinkle agent, which is encapsulated in nanosomes. Nanosomes penetrate deep into the epidermis to work at the heart of wrinkles.

Revitalift Double Lifting	Antiwrinkle	L'Oreal	It contains nanosomes of Pro-Retinol A. RevitaLift Double Lifting is a unique dual action treatment that instantly retightens skin and effectively fights wrinkles.

Eye Tender	Antiwrinkle	Kara Vita	It contains nanospheres, delivers 13 bioactives including proven, wrinkle-reducing peptides to stimulate fibroblasts, build collagen, brighten skin, and reduce inflammation for a younger, healthier appearance.

Eye Contour Nanolift	Antiwrinkle Antiaging	Euoko	It is based on nanocapsules technology. Lifting nanocapsules join seven other immediate and long-term fighters of fine lines, wrinkles, and puffiness. It provides instant and long-term smoothness, gives the eye area more radiance, and diminishes the appearance of dark circles and puffiness.

Soleil Soft-Touch Anti-Wrinkle Sun Cream SPF 15	Antiwrinkle sunscreen	Lancôme	It contains vitamin nanocapsules which help to preserve skin's youth effectively. SPF 15 offers optimal protection against the sun. It contains exclusive ingredients to guarantee a long-lasting effect.

Nano Gold Firming Treatment	Antiaging	Chantecaille	Infinitely small nanoparticles of pure gold are bound to silk microfibers to firm and tone skin, while delivering incredible anti-inflammatory, healing, and age defying power.

Nanosphere Plus	Antiaging	DermaSwiss	A stem cells revolutionary antiaging therapy Nanosphere Plus serum has been specially formulated to allow natural stem cells to preserve and protect skin cells. Using the cells from a rare Swiss apple (Uttwiler Spatlauber), Nanosphere Plus protects longevity and combats chronological aging.

Zelens Fullerene C-60 Night Cream	Antiaging	Zelens	Fullerene C-60 is a naturally occurring microscopic form of carbon which was found to have remarkable antioxidant properties.

Clearly It! Complexion Mist	Antiacne	Kara Vita	This nanosphere technology-based product tackles acne conditions and balances sebum production. Nanosphere time-released bioactives stimulate capillary activity for all-day detoxifying results.

DiorSnow Pure UV Base SPF 50	Sunscreen	Dior	Contains nano-UV filters for ultraprotection against the damaging effects of UVA and UVB rays.

Soleil Instant Cooling Sun Spritz SPF 15	Sun protection spray	Lancôme	Contains vitamin nanocapsule. Instant cooling sun spray SPF 15 immediately offers a sensation of freshness. SPF 15 provides optimal protection against the sun.

Fresh As A Daisy Body Lotion	Body lotion	Kara Vita	This lotion uses nanospheres to quickly penetrate, moisturize, and nourish all types of skin.

Cosil Nano Beauty Soap	Cleanser	Natural Korea	Silver nanoparticles are highly effective as disinfectant and guarantee protection of skin.

Cosil Whitening Mask	Face mask	Natural Korea	Made with nanocolloidal silver used for the effect of getting rid of germs from your face, compressing pores, soothing the skin condition, and keeping your skin radiant and soft.

Nanorama—Nano Gold Mask Pack	Face mask	LEXON NanoTech	It contains pure nanosized gold that is highly effective in penetrating small pores and disinfecting skin, helps to reduce pore size, and prevents and treats acne. It is well known that nanogold is very effective disinfectants.

Primordiale Optimum Lip	Lip treatment	Lancôme	Delivers 100% botanically pure vitamin E via nanocapsule technology to reduce lip bleeding and feathering due to fine lines and wrinkles.

Lip Tender	Lip moisturizer	Kara Vita	Ten bioactive ingredients are precisely calculated to work within lyphazomes, delivering a 4-in-1 formula and bringing long-lasting hydration for fast and dramatic lip repair.

Nano Cyclic Cleanser Silver	Cleanser	Nano Cyclic	Cyclic cleanser is a scientifically balanced blend of nanosilver and natural ingredients. It kills harmful bacteria and fungi, treats acne, exfoliates dead skin on all parts of the body, diminishes age spots, deodorizes the body, and fights wrinkles.

LifePak Nano	Face gel	Pharmanex	LifePak Nano is a nutritional antiaging program formulated to nourish and protect cells, tissues, and organs in the body with the specific purpose of guarding against the ravages of aging. Nanoencapsulation increases bioavailability coenzyme Q10 by 5–10 times.

**Table 2:** Patent review on nanotechnology-based cosmeceuticals.

Title	Publication number	Publication date	Applicant
Cosmetic composition containing retinol stabilized by porous polymer beads and nanoemulsion	EP 2583665A2	April 24, 2013	Act Co., Ltd
Multiactive microtargeted antiaging skincream polymer technology	EP 20110798597	April 17, 2013	NY Derm LLC
Semipermanent mascara and method of applying	US 20130068242A1	March 21, 2013	Cry Baby Culture
Topically administered, skin-penetrating glycosaminoglycan formulations suitable for use in cosmetic and pharmaceutical applications	US 20130059769A1	March 7, 2013	Eva Turley
Biodegradable, biocompatible, and nontoxic material sheets consisting of said material and the use thereof in food, pharmaceutical, cosmetic, and cleaning products	US 20130034638A1	February 7, 2013	Inis Biotech LLC
Metal oxide nanocomposites for UV protection	US 20130022655A1	January 24, 2013	BASF SE
Oil-in-water-type emulsion sunscreen cosmetic composition	US 20130011348A1	January 10, 2013	Tomiko Takakura
Synthetic collagen threads for cosmetic uses including skin wrinkle treatments and associated methods	US 20130018415A1	January 17, 2013	Rebeccah Brown
Deodorant composition	WO 201210122A1	August 2, 2012	Ilios Srl
Preparation of cationic nanoparticles and personal care compositions comprising said nanoparticles	EP 2254545A2	December 1, 2010	BASF SE
Gel technology suitable for use in cosmetic compositions	US 20100266649A1	October 21, 2010	Avon Products, Inc.
Nanocrystals for use in topical cosmetic formulations and thereof method of production	EP 2099420A1	September 16, 2009	Abbott GmbH & Co.KG
Nanodiamond UV protectant formulations	US 20090220556A1	September 3, 2009	International Technology Center
Nanoparticle compositions providing enhanced color for cosmetic formulations	US20090175915A1	July 3, 2009	Avon Products, Inc.
Nanocomposite pigments in a topical cosmetic application	WO 2008079758A1	July 3, 2008	Avon Products, Inc.
Cosmetic pigment composition containing gold or silver nanoparticles	EP 1909745A1	April 16, 2008	Korea Research Institute of Bioscience and Biotechnology
Antimicrobial silver compositions	WO 2006026026A2	March 9, 2006	Acry Med, Inc.
Long-lasting coatings for modifying hard surfaces and processes for applying the same	US 6955834B2	October 18, 2005	The Proctor & Gamble Company
Nail polish compositions comprised of nanoscale particles free of reactive groups	US 20050220730A1	October 6, 2005	Martinez Francisco
Use of nanoscale deodorants	EP 1239823B1	June 16, 2004	Cognis Deutschland GmbH & Co.KG
Cosmetic compositions comprising nanoparticles and processes for using the same	US 20030064086A1	March 13, 2003	Danuvio Carrion
A controlled delivery system for hair care products	WO 2002060399A1	August 8, 2002	Salvona LLC
Antimicrobial body care product	WO 2000078281A1	December 28, 2000	Bernhard Hanke

**Table 3:** Research studies done on percutaneous absorption of TiO_2_ and ZnO nanoparticles.

Test material	Skin model	Particle size	Results	Reference
TiO_2_	Dermatomed skin	1–35 nm, (noncoating), 2–35 nm (alumina/silica/silicon coated), and 3–10 × 100 nm (mixture of alumina and silicon coated)	No penetration was observed regardless of TiO_2_ type in intact and stripped skin. SEM-EDS observation showed that Ti penetrated into vacant hair follicles (greater than 1 mm below the skin surface); however it did not penetrate into dermis and viable epidermis.	[70]

TiO_2_	Human skin *in vitro *	20 nm	Penetration in restricted to the topmost corneocyte layers in the stratum corneum. No penetration into living skin was observed.	[71]

TiO_2 _alone or in combination with ZnO	Human skin (biopsy)	20 nm	TiO_2_ or ZnO nanoparticles are absent or their concentration is too low to be tested under the stratum corneum in human viable epidermis. Therefore, significant penetration towards the underlying keratinocytes is unlikely.	[72]

TiO_2_ in a sunscreen formulation	Human skin *in vitro* and human subjects	20 nm	Results showed penetration is limited to upper layers of stratum corneum. No penetration in skin furrows or follicular opening may be mistaken for penetration in the epidermal compartment.	[73]

TiO_2_	Human skin *in vitro *	10 nm to 100 nm	Results showed penetration of particles into the upper layers of stratum corneum. No penetration into living skin.	[74]

TiO_2_ in various formulations	Pig skin *in vitro *	Needles: 45 to 150 nm × 17 to 35	Particles on/in the stratum corneum; minimal penetration into stratum granulosum. No penetration into living skin.	[75]

TiO_2_	Human subjects (biopsy)	150 nm to 170 nm	Results showed particles in the upper layers of stratum corneum. About 1% of particles in the follicle ostium. No penetration into living skin.	[76]

TiO_2_ and ZnO	Human skin *in vitro *	TiO_2_: 50 to 100 nm ZnO: 20 to 200 nm	Results showed that penetration is limited to upper layers of stratum corneum.	[77]
